# Spindle-E Acts Antivirally Against Alphaviruses in Mosquito Cells

**DOI:** 10.3390/v10020088

**Published:** 2018-02-18

**Authors:** Margus Varjak, Isabelle Dietrich, Vattipally B. Sreenu, Bethan Eluned Till, Andres Merits, Alain Kohl, Esther Schnettler

**Affiliations:** 1 MRC-University of Glasgow Centre for Virus Research, Glasgow G61 1QH, UK; isabelle.dietrich@ndm.ox.ac.uk (I.D.); Sreenu.Vattipally@glasgow.ac.uk (V.B.S.); btill@harper-adams.ac.uk (B.E.T.); alain.kohl@glasgow.ac.uk (A.K.); 2 Institute of Technology, University of Tartu, Nooruse 1, Tartu 50411, Estonia; andres.merits@ut.ee; 3 Bernhard-Nocht-Institute for Tropical Medicine, Bernhard-Nocht-Strasse 74, Hamburg 20359, Germany; 4 German Centre for Infection Research (DZIF), partner site Hamburg-Luebeck-Borstel-Riems, Hamburg 20359, Germany

**Keywords:** RNA interference, antiviral responses, *Ae. aegypti*, alphavirus

## Abstract

Mosquitoes transmit several human- and animal-pathogenic alphaviruses (*Togaviridae* family). In alphavirus-infected mosquito cells two different types of virus-specific small RNAs are produced as part of the RNA interference response: short-interfering (si)RNAs and PIWI-interacting (pi)RNAs. The siRNA pathway is generally thought to be the main antiviral pathway. Although an antiviral activity has been suggested for the piRNA pathway its role in host defences is not clear. Knock down of key proteins of the piRNA pathway (Ago3 and Piwi5) in *Aedes aegypti*-derived cells reduced the production of alphavirus chikungunya virus (CHIKV)-specific piRNAs but had no effect on virus replication. In contrast, knock down of the siRNA pathway key protein Ago2 resulted in an increase in virus replication. Similar results were obtained when expression of Piwi4 was silenced. Knock down of the helicase Spindle-E (SpnE), an essential co-factor of the piRNA pathway in *Drosophila melanogaster*, resulted in increased virus replication indicating that SpnE acts as an antiviral against alphaviruses such as CHIKV and the related Semliki Forest virus (SFV). Surprisingly, this effect was found to be independent of the siRNA and piRNA pathways in *Ae. aegypti* cells and specific for alphaviruses. This suggests a small RNA-independent antiviral function for this protein in mosquitoes.

## 1. Introduction

In the last decade chikungunya virus (CHIKV; family *Togaviridae*, genus *Alphavirus*) has caused epidemic outbreaks in Africa, Asia, and more recently the Americas as well as sporadic outbreaks in Europe [1]. The symptoms are high fever, joint pain, and rash; often chikungunya infection also causes long term chronic diseases [1,2,3]. CHIKV is transmitted by the *Aedes albopictus* and *Ae. aegypti* mosquitoes; the latter is also an important vector for other arboviruses in the *Flaviviridae* family like the dengue virus (DENV; genus *Flavivirus*) and Zika virus (ZIKV; genus *Flavivirus*) [1]. Arbovirus infections in mosquitos and mosquito cell cultures are believed to be mainly asymptomatic and persistent, possibly due to an effective innate immune responses including the RNA interference (RNAi) pathway [4,5]. Of these, the short-interfering (si)RNA pathway is considered to be the main antiviral mechanism. It is initiated by virus-specific double-stranded (ds)RNA cleavage carried out by Dicer-2 (Dcr2), producing 21 nt long dsRNA fragments called siRNAs which are loaded into Argonaute-2 (Ago2), a member of the RNA-induced silencing complex (RISC) [4,5]. In RISC, one of the complementary strands of the virus-specific siRNA (vsiRNAs) is degraded and the remaining strand directs Ago2 to a complementary (viral) RNA target, resulting in (viral) RNA cleavage and degradation [6,7,8]. Knock down and knock out experiments have shown that Dcr2 and Ago2 are antiviral against various arboviruses in mosquitoes [9,10,11,12,13,14,15].

In addition to vsiRNAs, virus-specific PIWI-interacting (pi)RNAs have been reported in mosquitoes (and mosquito-derived cell lines) after infection by viruses of all major arbovirus families [14,16,17,18,19,20,21,22,23]. piRNAs are 24–29 nt in length and produced in a Dicer-independent manner. Most knowledge of the piRNA pathway is based on the *Drosophila melanogaster* model where it is mainly active in germ line cells controlling transposons [24]. In contrast, in mosquitoes piRNAs are also produced in somatic cells and can be transposon-, gene- or virus-specific [19,25,26]. Based on the *D. melanogaster* ping-pong piRNA production model, primary piRNAs are produced initially from long RNA transcripts transcribed from “piRNA clusters” in heterochromatin; following cleavage primary piRNAs with uridine at position 1 (U1) are produced and bound by Piwi or Aubergine (Aub) proteins [24,27]. Primary piRNAs target sequence-specific transposon-based RNA, resulting in the production of secondary piRNAs with adenine at position 10 (A10) and bound to Ago3. In turn, these secondary piRNAs target antisense RNAs, resulting in the production of primary-type secondary piRNAs (again bound to Piwi or Aub) [28]. Although ping-pong amplification of transposon based piRNAs have been reported in *Ae. aegypti*, differences in the key proteins of the piRNA pathway have been observed between the fly and *Ae. aegypti*, possibly due to expansion in the Piwi protein family in *Ae. aegypti* [14,25].

It is currently not known what initiates the production of virus-specific piRNA (vpiRNA) in mosquitoes and their exact role in the control of virus replication. Previous studies indicated that, at least in the case of the Sindbis virus (SINV; family *Togaviridae*, genus *Alphavirus*) and DENV, Ago3 and Piwi5 (and to a lesser extent Piwi6) are responsible for the production of vpiRNAs. The antiviral potentiality of piRNAs has been studied with the Semliki Forest virus family (SFV; *Togaviridae,* genus *Alphavirus*); knock down of Ago3 and Piwi5 results in the disappearance of SFV specific piRNAs [14,16,29], but it does not enhance SFV replication [14,29]. Similarly, a study conducted with the mosquito-borne Bunyamwera virus (BUNV; family *Peribunyaviridae*, genus *Orthobunyavirus*) indicated no antiviral activity of Ago3, Piwi5 and Piwi6 [20]. In contrast, Piwi4 has been reported as antiviral protein against SFV, BUNV, ZIKV and Rift Valley fever (RVFV, family *Phenuiviridae*, genus *Phlebovirus*), though it does not bind piRNAs [16,21,23,29] and is not required for the production of SFV-, SINV- or DENV-specific piRNAs [14,16,30].

In addition to these key RNAi effector proteins, other proteins are assumed to play important roles as co-factors of these pathways. The helicase Spindle-E (SpnE) has been shown to be essential for piRNA-mediated transposon silencing in *D. melanogaster* and required for the ping-pong amplification of piRNAs. It physically interacts with different piRNA pathway proteins, including Aub, Ago3 and Qin and thereby acts as a co-factor in the ping-pong amplification loop [31,32,33,34,35]. Moreover, mutant SpnE flies have an enhanced West Nile virus (WNV; family *Flaviviridae,* genus *Flavivirus*) replication and in these flies, drosophila X virus (family *Birnaviridae*, genus *Entomobirnavirus*) infection results in higher pathogenicity compared to control flies [36]. These results support an antiviral role of SpnE in the fly model; however, nothing is known about the possible antiviral role of SpnE in mosquitoes. Here, SpnE was found to be antiviral in *Ae. aegypti* derived cells but, surprisingly, the antiviral effects were limited to alphaviruses. Interestingly, the observed antiviral effect did not occur via the siRNA or piRNA pathways.

## 2. Materials and Methods

### 2.1. Plasmids and Viruses

Plasmids pIZ-Fluc and pAcIE1-Rluc for luciferase sensor assay have been described previously [29]. Plasmid pCMV-SFV4(3H)-FFLuc, contains reporter virus cDNA based on the SFV4 clone of SFV and has been described previously [29]. The reporter virus SFV4(3H)-FFLuc expresses Firefly luciferase as part of its non-structural polyprotein where the marker is located between nsP3 and nsP4 proteins and is flanked by duplicated nsP2 cleavage sites. Plasmid pCMV-SFV4 was used to rescue wild type SFV strain 4 [37]. CHIKV reporter virus CHIKV-2SG-FFLuc was rescued from cDNA plasmid pSP6-ICRES1-2SG-FFLuc. This construct is based on LR2006OPY1 stain of CHIKV belonging to East/Central/South African genotype [38], in this virus FFLuc is cloned between the non-structural and structural regions and expressed from a sub genomic promoter. Wild-type CHIKV was rescued from plasmid pSP6-ICRES1. Nanoluciferase-expressing BUNV (BUNV-NL) has been described previously [20]. The patient-derived isolate of ZIKV (strain PE243) has been described previously [39].

### 2.2. Cells

*Ae. aegypti*-derived Aag2 cells [20] (received from P. Eggleston, Keele University, Newcastle, UK), were grown in L-15+Glutamax (Thermo Fisher Scientific, Waltham, MA, USA) supplemented with 10% Tryptose Phosphate Broth (TPB, Thermo Fisher Scientific), 10% fetal bovine serum (FBS, Thermo Fisher Scientific), and penicillin-streptomycin (final concentration 100 units/mL, 100 µg/mL respectively, Thermo Fisher Scientific) at 28 °C. Baby hamster kidney (BHK-21) cells used for BUNV-NL, SFV4, SFV-(3H)-FFLuc, CHIKV or CHIKV-2SG-FFLuc rescue and titration were grown in GMEM (Thermo Fisher Scientific) supplemented with 10% TPB, 10% FBS and penicillin-streptomycin (final concentration 100 units/mL, 100 µg/mL, respectively) at 37 °C in 5% CO_2_ atmosphere. A549-Npro cells [39] (obtained from R. E. Randall, University of St. Andrews, St. Andrews, United Kingdom) were grown in DMEM (Thermo Fisher Scientific), supplemented with 10% FBS and used to grow and titrate ZIKV.

### 2.3. cDNA Synthesis and RT-PCR

Trizol (Thermo Fisher Scientific) was added to 1.5×10^5^ Aag2 cells per well of a 24-well plate, material from two wells was then combined to isolate total cellular RNA and purification conducted according to the manufacturer’s instructions. 1.5 µg of total RNA was used for cDNA synthesis using Superscript III reverse transcriptase (Thermo Fisher Scientific) and oligo(dT)15 primer (Promega, Madison, WI, USA). Semi-quantitative RT-PCR for *Ae. aegypti* transcripts was performed using gene-specific primers (Supplementary Table S1, in Supporting Information) and GoTaq DNA polymerase (Promega); actin transcripts were used as internal control.

### 2.4. Small RNA Sequencing and Sequence Analysis

Using Trizol reagent as per manufacturer’s protocol total RNA was extracted from 2×10^6^ virus infected Aag2 cells grown in T-25 flask. Small RNAs of 15–40 nt in length were gel purified and sequenced on an Illumina HiSeq 4000 at BGI Tech (Hong Kong, China). Obtained sequence reads were aligned to the SFV genome using in-house established BLAST guided bioinformatics pipeline, maximum of one mismatch or indel was allowed in the alignments. Reads that matched to the reference genome with 18 bp to 35 bp in length were further analysed. These were separated into two groups: positive and negative based on their alignment to the genome and antigenome, respectively [23]. Small RNA sequencing data is available upon request.

### 2.5. Small RNA Analysis by Northern Blot

Northern blot analysis was conducted as described previously [29,40]. In short, total RNA was isolated from the SFV or CHIKV infected Aag2 cells (2×10^6^ cells, MOI 10, 48 hpi) by using Trizol reagent. 15 µg of RNA was size-fractioned on a 0.5× TBE, 7 M Urea, 15% polyacrylamide gel, thereafter transferred to Hybond NX nylon membranes (GE Healthcare, Chicago, IL, USA). RNA was chemically cross-linked to membrane by using 1-ethyl-3-(3-dimethylaminopropyl) carbodiimide (Sigma, St. Louis, MO, USA). SFV or CHIKV-specific small RNAs were probed with a set of DNA oligonucleotides (Supplementary Table S1) that were 5′ end-labelled with [^32^P] γ-adenosine-triphosphate (Perking Elmer, Waltham, MA, USA) using T4 Polynucleotide kinase (New England Biolabs, Ipswich, MA, USA). Hybridization of membranes with oligonucleotide-probes was performed overnight at 42 °C in Ultrahyb Oligo hybridization buffer (Thermo Fisher Scientific). Membranes were washed twice at 42 °C with each of the following three buffers: 2× SSC and 0.5% SDS, 2× SSC and 0.2% SDS, 0.2× SSC and 0.2% SDS. The membrane was exposed to a phosphoimager screen for signal detection and quantification.

### 2.6. Transfection of Nucleic Acids and Virus Infections

For transfections, 1.5×10^5^ Aag2 cells were seeded per well of a 24-well plate and were transfected using 2 µL of Dharmafect 2 reagent (GE Healthcare). To silence host transcripts, 300 ng of gene-specific dsRNAs per well were transfected into Aag2 cells, followed by virus infections at 24 h post-transfection (pt). Alternatively, for RNAi reporter assays, 24 h pt 10 ng of dsRNA (FFLuc specific or mCherry specific) or 1 ng siRNA (targeting FFLuc or Hygromycin B resistance gene) were co-transfected with 50 ng of pIZ-Fluc and 50 ng of pAcIE1-Rluc plasmids; the FFLuc and Rluc activities were measured at 24 h pt. For small RNA sequencing analysis or northern blot analysis, Aag2 cells were grown in T-25 flask and transfected with 3 µg of gene-specific dsRNA using 10 µL of Dharmafect 2, followed by virus infection at 24 h pt.

### 2.7. dsRNA Production

Gene-specific primers flanked by T7 RNA polymerase promoter sequences were used to amplify unique gene-specific fragments that were validated through Sanger sequencing. PCR products were then used for in vitro transcription using the RNAi Megascript kit (Thermo Fisher Scientific) according to manufacturer’s instructions. The obtained RNAs were treated with Dnase1 and RNaseA and purified using columns provided with the kit. Previously described and verified dsRNAs were used for silencing of Ago2, Piwi4, Piwi5, Piwi6, Ago3, mCherry and eGFP [14,20,21,29]; alternatively new silencing dsRNAs were developed (see Supplementary Table S2).

### 2.8. Luciferase Assays

Cells were lysed with passive lysis buffer (Promega) and Firefly luciferase activity measured by using the Luciferase Assay System (Promega) for SFV4(3H)-FFLuc, the Steady-Glo luciferase assay (Promega) for CHIKV-2SG-FFLuc and the Nano luciferase assay (Promega) for BUNV-NL infected cells. To measure both Firefly and *Renilla* luciferases in RNAi sensor assays, the Dual Luciferase Reporter Assay System (Promega) was utilised. Luciferase activities were determined in a Glomax luminometer.

## 3. Results

Since Piwi4 acted antivirally against different arboviruses, we hypothesized that other proteins possibly involved in the piRNA pathway could be antiviral, too [14,21]. To determine the antiviral activity of these proteins in mosquito cells, dsRNA-based knock down experiments can be used to determine their effects on viral replication. Therefore, homologs of selected *D. melanogaster* proteins, known to act in different parts of the piRNA pathway, were identified in *Ae. aegypti* using the BLAST algorithm (Supplementary Table S2) [28,35,41,42,43,44,45,46,47,48,49]. In *D. melanogaster* Qin, Vasa and SpnE are needed for proper loading of the piRNAs during the ping-pong amplification between Piwi/Aub and Ago3. Zucchini (Zuc), Armitage (Arm) and GasZ is thought to be involved in the producing of primary piRNAs and trimming of piRNAs. Hen-1 carries out methylation of mature piRNAs at their 3′ termini [28,35,41,42,43,44,45,46]. Hp1a, Blm and Recq4 are involved in heterochromatin silencing, including the regions that contain “piRNA clusters” [14,47,48]. Mutations of Arm have led to increased susceptibility to viral infection in *D. melanoaster* [49]. To test gene expression of homologues of these candidates in Aedes aegypti, total cellular RNA was isolated from *Ae. aegypti*-derived Aag2 cell lines and subjected to cDNA synthesis. Gene-specific primers were used to verify the expression of the genes of interest, followed by sequencing verification. Afterwards, gene-specific primers with T7 RNA polymerase binding sides, were used to amplify a unique region of the transcript to obtain PCR fragments that were used to synthesize gene specific dsRNAs by in vitro transcription.

**Figure 1:**
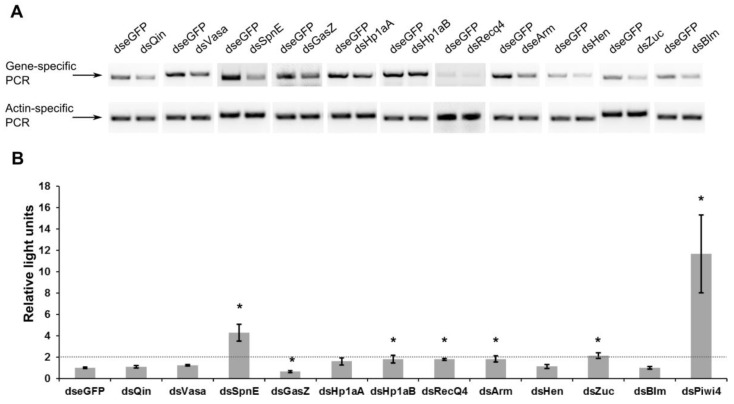
Effect of knock down of piRNA-related transcripts in Aag2 cells on SFV. Aag2 cells were transfected with unique, gene-specific dsRNAs against Piwi4 (positive controls) and potential piRNA pathway proteins. (**A**) Knock down of Ae. aegypti homologs of Drosophila transcripts of the piRNA pathway using dsRNA (dseGFP as control). Gene-specific primers were used to confirm silencing and an actin PCR product was used as internal control; (**B**) Aag2 cells transfected with gene specific dsRNAs (dsRNA against eGFP was used as negative control) were infected with FFLuc-expressing SFV (MOI 0.01) and cells lysed 48 hpi. FFLuc activities were measured and normalized to these from control cells (taken as 1); the dotted line represents the threshold level of at least a two-fold increase in luciferase levels. The mean values and standard error of three experiments conducted independently in triplicate are shown; * denotes *p* < 0.05 (according to Student’s *t*-test).

Following this, gene-specific dsRNAs or eGFP specific dsRNA (as control) were transfected to silence target transcripts (Figure 1A) in Aag2 cells. 24 h later, transfected cells were infected with firefly luciferase (FFLuc) expressing SFV4(3H)-FFLuc [29] at a multiplicity of infection (MOI) of 0.01. At 48 h post-infection (hpi), cells were lysed and FFLuc levels determined. Knock down of Piwi4 was used as positive control and confirmed the previously reported increase in luciferase levels [14]. From genes analysed in this experiment, transfection of SpnE-specific dsRNA, resulted in more than 2-fold increased luciferase levels compared to the control; supporting the antiviral activity of SpnE against SFV (Figure 1B). The effect of other gene knock down was less prominent or not significant. Interestingly, transfection of GasZ specific dsRNA was the only one that resulted in a decrease of luciferase expression.

**Figure 2:**
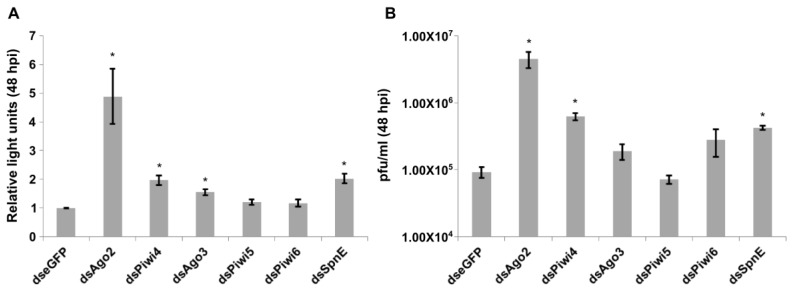
Effect of gene silencing on CHIKV replication. (**A**) Aag2 cells were transfected with dsRNAs to silence indicated transcripts, infected with CHIKV-2SG-FFLuc at 24 h pt, and lysed at 48 hpi. FFLuc activities were determined and are presented as relative mean luciferase activity values (normalized to activity in dseGFP treated). The error of the mean from three experiments conducted in triplicate are shown; (**B**) cells transfected as described above were infected with wt CHIKV at MOI of 0.01, and media was harvested 48 hpi, followed by plaque titration. The mean values of four independent experiments with standard deviation are shown. * indicates significance, *p* < 0.05 (Student’s *t*-test).

To determine if the antiviral effect of SpnE could be observed for a related alphavirus, similar knock down experiments were performed with CHIKV. Since less is known about the interactions of the mosquito RNAi response and CHIKV, it was important to perform some base line experiments with CHIKV in Aag2 cells. It was previously shown that CHIKV-infected cells and mosquitoes produced CHIKV specific siRNA and piRNAs, and that the majority of the vpiRNAs mapped to the final third of the genome; the region encoding for the structural proteins [19]. Moreover, knock down of the siRNA pathway key protein Ago2 resulted in an increased CHIKV replication in *Ae. aegypti*-derived cells and mosquitoes [11]. However, there are no data on the possible antiviral effects of Piwi proteins during CHIKV infection. To assess this, Aag2 cells were transfected with previously validated and tested dsRNAs [14,20,21,23] against eGFP (negative control), Ago2 (positive control), Piwi4, Ago3, Piwi5, Piwi6 and SpnE. Transfected cells were infected with FFLuc expressing CHIKV (CHIKV-2SG-FFLuc (Figure 2A) at MOI of 0.01 and luciferase levels determined at 48 hpi. At least 2-fold increases of luciferase levels were observed following knock down of Ago2 and Piwi4. The increase was smaller in Ago3 knock down cells and not significant upon knock down of Piwi5 or Piwi6. Moreover, knock down experiments with dsRNA against SpnE also showed an increase in luciferase levels; confirming previous results with SFV (Figure 2A). Similar results were observed for the production of infectious viral particles when wild type (wt) CHIKV was used (Figure 2B).

**Figure 3:**
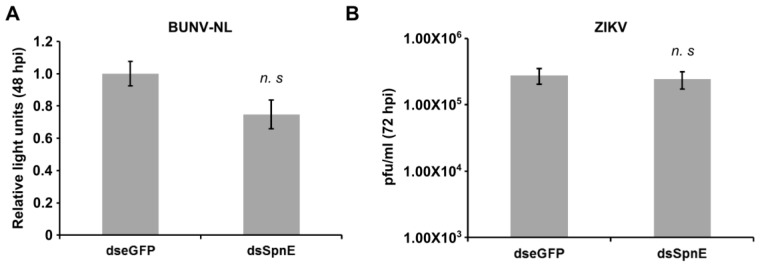
Effect of SpnE silencing on BUNV and ZIKV replication. dsRNAs were transfected into Aag2 cells to silence SpnE and cells treated with eGFP specific dsRNAs were used as control. At 24 hpt cells were infected with either Nanoluc-expressing BUNV-NL or wt ZIKV. (**A**) Cells infected with BUNV-NL (at MOI of 0.01) were lysed at 48 hpi; the relative mean luciferase activity values (normalized to these from dseGFP treated cells), together with the error of the mean from three independent experiments conducted in triplicate, are shown; (**B**) media was harvested at 72 hpi from wild-type ZIKV infected (MOI of 0.1) cells and subjected titration. The mean virus titre together with standard error of 6 independent experiments are shown. “n. s” indicates non-significance according to Student’s *t*-test.

To determine if the antiviral effect of SpnE is alphavirus-specific or could be broadened to arboviruses from other families, knock down experiments were repeated and cells infected with nanoluciferase reporter expressing BUNV-NL [20] or wild-type ZIKV [39]. No difference in nanoluciferase levels were detected in BUNV-NL infected cells (MOI 0.01) lysed 48 hpi (Figure 3A). Similarly, titration of ZIKV collected at 72 hpi (MOI 0.1) indicated that SpnE silencing had no beneficial effect (Figure 3B). In contrast, improved replication of BUNV-NL and ZIKV was observed in previous experiments (performed with the same experimental set up) for knock down of Piwi4 [20,23]. Thus, the results suggested that the antiviral activity of SpnE is alphavirus-specific.

It was previously suggested that SpnE affects viral replication by altering the siRNA response in *D. melanogaster* [36]. Moreover, SpnE has been reported to interact with proteins of the fly piRNA pathway and is critical for piRNA amplification [31,32,33,34,35]; however, piRNAs are not antiviral in flies [50].

To study the involvement of SpnE in the siRNA-based silencing in mosquitoes, luciferase sensor assays were carried out. In a first step, Aag2 cells were transfected with dsRNAs targeting eGFP (as negative control), Ago2 (positive control), Piwi4 or SpnE. In the second step, FFLuc expressing plasmid (pIZ-Fluc) and *Renilla* luciferase (Rluc) expressing construct (pAcIE1-Rluc, used as internal control), were co-transfected with siRNAs targeting FFLuc or Hygromycin B resistance gene (Hyg) (negative control).

**Figure 4:**
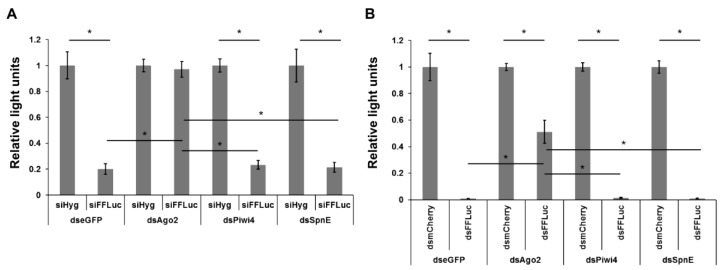
Effect of SpnE knock down on the siRNA pathway based silencing efficiency. Aag2 cells were first treated with dsRNA against eGFP (control), Ago2, Piwi4 or SpnE. 24 h later the cells were co-transfected with FFLuc and Rluc expressing reporter plasmids (pIZ-Fluc and pAcIE1-Rluc) and siRNAs against FFLuc (siFFLuc) or Hygromycin B (siHyg) (**A**) to assess the silencing ability of pre-formed siRNAs in the cells, or alternatively dsRNAs targeting FFLuc (dsFFLuc) or mCherry (dsmCherry) (**B**) to assess the silencing ability of siRNAs produced in a Dcr2-dependent manner. Cells were lysed at 24 hpt and activities of luciferases determined. Relative luciferase levels are shown on the Y-axis (with FFLuc/Rluc ratio in siHyg or dsmCherry transfected cells set to 1). The mean values with standard error are shown for three independent experiment conducted in triplicate, * shows *p* < 0.05 using two-way ANOVA.

Reduced levels of FFLuc were observed in dseGFP treated cells transfected with siFFLuc, compared to cells treated with siHyg (Figure 4A). As expected, knock down of the siRNA pathway key protein Ago2 resulted in reduced silencing efficiency of FFLuc-specific siRNAs (Figure 4A). At the same time, silencing of Piwi4 had no effect on FFLuc silencing; confirming previous results that Piwi4 did not act via the siRNA pathway [29] (Figure 4A). Similarly, SpnE silencing did not affect the efficiency of siRNA-based silencing of FFLuc in Aag2 cells (Figure 4A).

During virus infection vsiRNAs are produced from long dsRNAs by Dcr2-mediated cleavage, thus it was important to control whether silencing of SpnE could affect dsRNA processing into functional siRNAs. A dsRNA-based reporter assay was performed in Aag2 cells, similar to the previous described siRNA-based RNAi reporter assay. Therefore, Aag2 cells previously treated with dsRNA against Ago2, Piwi4, SpnE or eGFP (negative control) were co-transfected with pIZ-Fluc, pAcIE1-Rluc plasmids and dsRNAs targeting mCherry (negative control) or FFLuc. Again, knock down of Ago2 significantly decreases the dsRNA-based silencing of FFLuc gene (Figure 4B). In contrast, neither silencing of Piwi4 nor SpnE disrupted dsRNA-based silencing of FFLuc reporter (Figure 4B). These experiments suggest that SpnE did not affect the siRNA pathway in mosquito cells, and thus the antiviral activity of SpnE was not due to interactions with the siRNA pathway.

**Figure 5:**
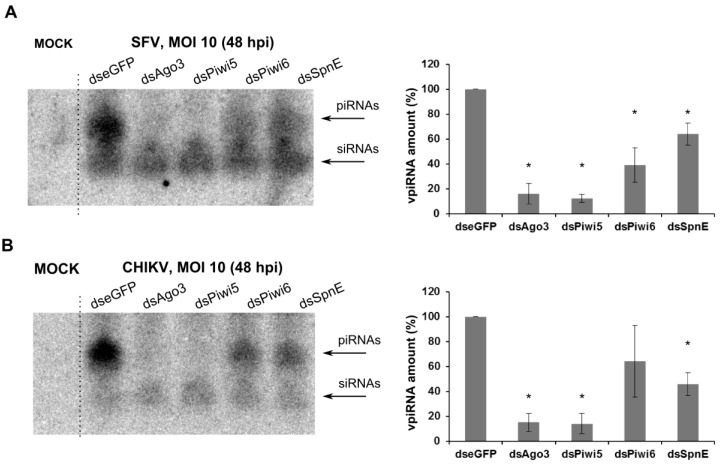
Northern blot analysis of virus-specific piRNAs. Aag2 cells were transfected with dsRNAs against Ago3, Piwi5, Piwi6, SpnE or eGFP (as negative control). 24 h pt the cells were infected with wt SFV (**A**) or wt CHIKV (**B**) at a MOI of 10. Total RNA isolated at 48 hpi was separated on PAGE-Urea gel, transferred onto nylon membrane and probed with virus specific radiolabelled DNA oligos for piRNAs and siRNAs. In the left panel, a representative image of three independent experiments is shown; in the right panel the mean levels of three independent experiments with standard deviation of SFV- or CHIKV-specific vpiRNAs are shown, * indicates significance, *p* < 0.05 (Student’s *t*-test); vpiRNA levels in control dseGFP treated cells taken as 100%.

To detect if SpnE affects the production of viral specific, vpiRNAs, Aag2 cells treated with dsRNAs against eGFP, Ago3, Piwi5, Piwi6 and SpnE were infected with SFV or CHIKV at a MOI of 10 and total cellular RNA isolated at 48 hpi. Total RNA was size-separated on gel, transferred onto Nylon membrane and probed for virus-specific small RNAs. As previously reported, silencing of Ago3 and Piwi5 resulted in almost complete disappearance of SFV-specific piRNAs, supporting their involvement in the SFV-specific vpiRNA production. In contrast, only a modest 60% or 30% reduction in SFV-specific piRNAs was detected in Piwi6 or SpnE silenced cells compared to control cells, respectively. Similar results were obtained with CHIKV, where knock down of Ago3 and Piwi5 resulted in nearly complete loss of vpiRNA production. Again, Piwi6 and SpnE knock down only diminished vpiRNA amounts by approximately 50% (Figure 5).

**Figure 6:**
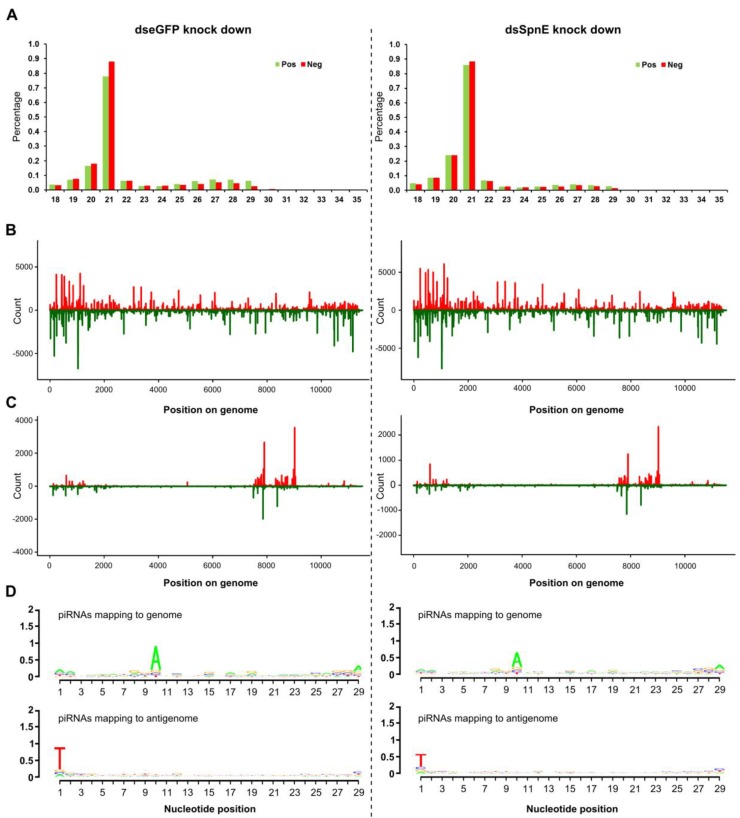
Analysis of SFV-specific small RNAs from Aag2 cells. dsRNAs were used to knock down eGFP (Left panel, as control) or SpnE (Right panel) in Aag2 cells. At 24 h pt cells were infected with SFV at an MOI of 10. At 48 hpi total RNA was isolated and small RNAs collected and sequenced. (**A**) Size distribution of small RNA sequences mapping to the SFV genome (red) or antigenome (green) (as percentage of total reads). Distribution of 21 nt (**B**) or 27 nt (**C**) long RNAs along the SFV genome (red, positive numbers on Y axis) or antigenome (green, negative numbers on Y axis); (**D**) relative nucleotide frequency and conservation per position of the 24- to 29-nt long piRNAs mapping to the SFV genome or antigenome; as DNA template was used for sequencing, U is represented by T.

The effect of SpnE silencing on total SFV specific vpiRNA levels and their distribution was further characterized by small RNA sequencing (Figure 6, Supplementary Table S3). Previous studies by us have indicated that SFV-specific vpiRNA production is dependent on Ago3 and Piwi5 [14,29]. Their knock down resulted in overall reduction in the number of sense and antisense strand-specific piRNAs, though there was no change in distribution along the genome/antigenome. Consistent with data from previous experiments, knock down of SpnE indeed reduced the total production of SFV-specific vpiRNAs (Figure 6A) to about 30% compared to the levels in control cells that were transfected with dsRNAs against eGFP (Figure 6A, Supplementary Table S3). No changes in the overall vsiRNAs amounts, nor their distribution along genome and antigenome (Figure 6A,B) were detected in SpnE silenced cells. Knock down of SpnE did not affect the vsiRNA distribution (Figure 6B), and despite the slightly reduced amounts of vpiRNAs, genome or antigenome mapping of these small RNAs was also unaltered (Figure 6C). vpiRNAs showed an A10 or U1 bias dependent on location on the genome or antigenome, respectively (Figure 6D).

Since the majority of piRNAs are transposon-specific, we tested if SpnE can affect their levels. We focused our analysis on the top 50 piRNA producing transposonsable elements (TE) according to Miesen *et al.* [16]. In our dataset piRNAs could be mapped to all 50 transposons, however, the number of reads for two of them was too low, thus these transposon-specific piRNAs were excluded. The majority of transposon-specific piRNAs were of antisense polarity (Supplementary Table S4) and 18 transposons produced enough sense strand specific reads for further analysis. These results indicated that on average SpnE knock down had no effect on total piRNA production. However, SpnE silencing reduced the sense strand specific piRNAs for 5 of the studied transposons, though the reduction was moderate (between 30–45%). Overall, these results suggest that the alphavirus-specific antiviral activity of SpnE is not directly linked to the small RNA pathways nor that SFV- or CHIKV-specific piRNAs are antiviral in the performed experimental set up.

## 4. Discussion

Initially in *D. melanogaster,* SpnE was found to be required for microtubule network formation, RNA localization and embryonic pattern formation, later it was also found to be associated with the piRNA pathway, SpnE has DExH helicase and Tudor domains and in *D. melanogaster*, SpnE has been shown to associate with Vasa, Qin, Aub and Ago3 where it is involved in the ping-pong amplification loop of transposon-specific piRNAs [31,32,33,34,35,51]. Moreover, SpnE cycles between the cytoplasm and the so-called nuage structures. Nuage structures are close to the cytoplasmic face of nuclear pores and are sites where the ping-pong cycle occurs and TE silencing takes place. These reports indicated that SpnE was not required for nuage assembly and that SpnE was required for maintenance of Aub and Ago3 protein levels, but not for transcription of these genes [35,52]. In *D. melanogaster* mutant for SpnE WNV replication increased compared to control flies which suggested an antiviral activity of SpnE in this model via a RNAi-dependent mechanism; however, this was not directly shown [36].

Little is known about the role of piRNA pathway proteins in mosquitoes. In contrast to *D. melanogaster* which express Piwi, Aub and Ago3, *Ae. aegypti* lacks Aub, but encodes Ago3 and an expansion of PIWI proteins (1-7 PIWI proteins) [14,25]. In the fly model piRNAs are produced only in germ line or ovarian sheet cells [53], whereas in mosquitoes piRNAs are also produced in somatic tissues [19]. Moreover, piRNAs in *D. melanogaster* appear not to act antivirally [50]. In the current study, it was established that infected mosquito cells produce CHIKV-specific piRNAs in a similar fashion to previously studied alphaviruses: SFV and SINV [14,16,29]. Knock down of Ago3 or Piwi5 results in disappearance of vpiRNAs, supporting their role in the ping-pong based piRNA production. However, as previously shown for SFV, knock down of Ago3 and Piwi5 had no significant effect on CHIKV infection. In contrast, knock down of Piwi4 resulted in increased CHIKV replication, similar to previous reports for other arboviruses, including BUNV, SFV and ZIKV [14,20,21,23]. However, Piwi4 does not bind piRNAs at all nor is it involved in SINV- or SFV-specific piRNA production [16,26,29].

We conducted a screen where we silenced—by using gene-specific dsRNAs—potential piRNA pathway proteins in Aag2 cells. With any knock down experiment there is likelihood of false negatives, and reduced RNA levels may not result in reduced protein levels. Regardless, knock down of SpnE resulted in increased replication of CHIKV and SFV, but not of BUNV and ZIKV, indicating that SpnE acts as an alphavirus specific antiviral protein in mosquito cells. Its mode of action is still unclear; however, the effects were not directly linked to the siRNA pathway. Neither is its antiviral activity strongly linked to the vpiRNA production; although it should be noted that SpnE (like Piwi6) knock down partly reduced SFV and CHIKV specific piRNA levels. Yet, almost complete loss of vpiRNA production (if Ago3 and Piwi5 were silenced) did not benefit SFV replication [14,29] or CHIKV replication (current study). Moreover, knock down of SpnE only affected a small number of (tested) transposon-specific piRNA.

Taken together the results indicate that SpnE is an antiviral protein, specifically for alphavirus infected mosquito cells and its activity is not mediated by the siRNA or piRNA pathway. More research is needed to understand the precise antiviral mode of action of SpnE in *Ae. aegypti*. SpnE is one of the proteins involved in the RNA metabolism and it may be that the antiviral activity is linked to such pathways, rather than RNAi.

## Supplementary Materials

The following are available online at http://www.mdpi.com/1999-4915/10/2/88/s1. Table S1: Oligos for northern blot. Table S2: Primers sequences. Table S3: Small RNA sequencing reads. Table S4: Transposon-specific small RNA reads
