# Supplementary material for: Spindle-E Acts Antivirally Against Alphaviruses in Mosquito Cells
Source: Viruses. 2018 Feb 18;10(2):88. doi: 10.3390/v10020088 (PMC5850395; doi:10.3390/v10020088)
Supplement: Supplementary file 1 [file viruses-10-00088-s001.pdf]

Supplementary Materials:

Table S1. Oligos for norther blot. Oligos used to detect SFV- or CHIKV-specific siRNAs and piRNAs by northern blot analysis.

**SFV siRNA-specific oligos**	GGTGCCCGACATGTCTGGAAA<br>CTTCAGCATCCCGTCGGTAGC<br>TTCTGACACCTTTCATCGCCT<br>GTTAAACTCTGAAGGCACCTT<br>AGGTCAATGATGGTCGGCGCC
**SFV piRNA-specific oligos**	AAGCTAGCTTTGCCAGGTCCGCGTTGT<br>ACGTTTCCGGTTCCACAGGTGCAGTTGTA<br>GTGCCAACGTTTCCGGTTCCACAGGTG<br>TCCGCGTTGTCGATGACTCCTTTCACG<br>TGTTCCGTTTCGGCACGTCAAGGCTGCC<br>TATCCTCGAGCATCCGTAGTGTGGCCTC
**CHIKV siRNA-specific oligos**	GCCATACAGATGCGGACCCAA<br>GCTTGCAGAGTGGCAGACCCG<br>GATACCCAGTCAATTCAGAAG<br>GGGCGTCAGCATCCAGGTCTG<br>CCGAGTGCTGGAAGATCGTCT<br>GCAGATATCTACAAACGGTAC<br>CCTCTCTGTCTCATCACGTCG<br>GCGTCTATGTCCACGTACACA<br>GCAGAGTAGAGCAGTAAGAAA<br>ACGTCCCTTTTCATATCTACT<br>TGTTCTACACAAGTACACTGT
**CHIKV piRNA-specific oligos**	GGATGAAGTGTCCCATTGTTCCAGTA<br>GCACGAGTGCCCTTCTCCACAGTCG<br>GCATCAGAAATGAAGCGACGGACGGG<br>ACCTGGATTTTCAGCGTCCCGTCTGT<br>GCTGTCATCCGTCTTTATTCCGATT<br>GGTCCAATCGTGGCTGTCATCCGTCT<br>GTTGTCGAAGATCGGTCTGCCGCTGTC<br>CCACGCGTCCCTTGTTGTCGAAGAT<br>ATTTTCGATTTTCATGCACATCCTC<br>GCCAGTTTGGCCAGGTCCGCGTTAT<br>CAGGTCTCATGACGTTGTCCTCAAG

Table S2. Primer sequences. Primers used to control gene expression and synthesize *T7 RNA polymerase promoter*-flanked PCR products for dsRNA synthesis.

**Gene name in Drosophila**	**Used Gene name for Ae aegypti**	**Accession number**	**Forward/ reverse primer sequences**
Armitage	Arm	AAEL010696	GTAATACGACTCACTATAGGGGTACCGTTGACATTGCTTCTGAAA/ GTAATACGACTCACTATAGGGTGCTAGGAACGGATCGATAATTGT
Spindle-E	Spindle-E	AAEL013235	GTAATACGACTCACTATAGGGGATAAATTCCGAACTCATCCGGCT/ GTAATACGACTCACTATAGGGGACAGAAGATCAGTGCCATCAGAG
GasZ	GasZ	AAEL000289	GTAATACGACTCACTATAGGGAACTGTAAGGACTCGTATGGGATG/ GTAATACGACTCACTATAGGGCGCTCGAAGGGTAATTTAAACTCC
Zuc	Zuc	AAEL011385	GTAATACGACTCACTATAGGGCTACCTAATGCACCACAAGTTCTG/ GTAATACGACTCACTATAGGGCGGAGTTATCAGCAGTGGATGTT
Hen1	Hen1	AAEL010029	GTAATACGACTCACTATAGGGTGAAATACTTTGGAATCGGGCCTC/ GTAATACGACTCACTATAGGGATCGTCGTCCCTCAAATCATACTC
Hp1a	Hp1aA	AAEL004467	GTAATACGACTCACTATAGGGAGTTGTCAATGGAAAGGTGGAGTA/ GTAATACGACTCACTATAGGGATAGTCGTCCTTTGCATCGTTCTT
Hp1a	Hp1aB	AAEL004484	GTAATACGACTCACTATAGGGGCAAAGCTAAGGATAACAAACCGA/ GTAATACGACTCACTATAGGGGAACAGAAGCTCGTTGTTTGCTT
Blm	Blm	AAEL004039	GTAATACGACTCACTATAGGGACATCTCGCCTATTAGCAAGACAT/ GTAATACGACTCACTATAGGGATCTGGTTTGCTTTGAATCTGGTG
RecQ4	RecQ4	AAEL010905	GTAATACGACTCACTATAGGGTGTCAGTTTGTGTGGTTTATTGGG/ GTAATACGACTCACTATAGGGTTGGCCGTCGTATTGTTATGATTG
Qin	Qin	AAEL014694	GTAATACGACTCACTATAGGGGCTCAACCTCATCAAGAACGTTA/ GTAATACGACTCACTATAGGGAATGACCAGATCTTGTCGGAGTA
Vasa	Vasa	AAEL004978	GTAATACGACTCACTATAGGGCACGAAACCTACTCCGATTCAAA/ GTAATACGACTCACTATAGGGATCATCTTTTCAACCGATGGCAT

Table S3. Small RNA sequencing reads. Number of SFV-specific sequencing reads obtained by analysing small RNA in total cellular RNA samples isolated from SFV-infected cells (MOI of 10) Aag2 cells. 24 h before infection the cells had been treated with dsRNA against eGFP or SpnE.

**Analysis of total cellular RNA from SFV-infected Aag2 cells if eGFP or SpnE had been knocked down**			
**Target**	**Number of SFV-specific siRNA reads**	**Number of SFV-specific 24-29 nt long reads**	**Number of total reads (18-35nt)**
dseGFP, replicate 1	737975	182025	50444460
dseGFP, replicate 2	829610	278988	50063949
dsSpnE, replicate 1	663639	128353	36243946
dsSpnE, replicate 2	1082618	203951	62102991

Table S4. Transposon-specific small RNA reads. Transposon-specific piRNA reads as a percentage of total reads (18-35nt) are shown

Transposon name	dseGFP knock down, replicate 1			dseGFP knock down, replicate 2			dsSpnE knock down, replicate 1			dsSpnE knock down, replicate 2			dseGFP knock down, average of two replicates			dsSpnE knock down, average of two replicates			Ratio (dsSpnE/dseGFP)		
	Sense and antisense specific	Sense-strand specific	Antisense-strand specific	Sense and antisense specific	Sense-strand specific	Antisense-strand specific	Sense and antisense specific	Sense-strand specific	Antisense-strand specific	Sense and antisens e specific	Sense-strand specific	Antisense-strand specific	Sense and antisense specific	Sense-strand specific	Antisense-strand specific	Sense and antisense specific	Sense-strand specific	Antisense-strand specific	Sense and antisense specific	Sense-strand specific	Antisense-strand specific
CR1_Ele9	0.148	0.004	0.144	0.157	0.003	0.154	0.148	0.002	0.145	0.158	0.002	0.155	0.153	0.004	0.149	0.153	0.002	0.150	1.000	0.628	1.009
CR1_Ele10	0.033	0.001	0.032	0.020	0.001	0.019	0.027	0.001	0.026	0.015	0.000	0.015	0.027	0.001	0.026	0.021	0.001	0.020	0.786	0.553	0.796
CR1_Ele22	0.010	0.000	0.010	0.008	0.000	0.008	0.009	0.000	0.009	0.007	0.000	0.007	0.009	0.000	0.009	0.008	0.000	0.008	0.892	0.230	0.894
Pao_Bel_Ele6	0.000	0.000	0.000	0.000	0.000	0.000	0.000	0.000	0.000	0.000	0.000	0.000	0.000	0.000	0.000	0.000	0.000	0.000	1.061	0.921	1.159
Pao_Bel_Ele28	0.045	0.009	0.036	0.034	0.005	0.028	0.047	0.009	0.038	0.034	0.005	0.029	0.039	0.007	0.032	0.040	0.007	0.033	1.024	1.006	1.028
Pao_Bel_Ele113	0.041	0.000	0.041	0.030	0.000	0.030	0.047	0.000	0.047	0.031	0.000	0.030	0.035	0.000	0.035	0.039	0.000	0.039	1.092	1.988	1.091
Pao_Bel_Ele179	0.415	0.000	0.415	0.558	0.000	0.558	0.488	0.000	0.487	0.588	0.000	0.588	0.487	0.000	0.486	0.538	0.000	0.538	1.105	0.657	1.106
Penelope_Ele6	0.089	0.002	0.086	0.118	0.003	0.115	0.109	0.003	0.105	0.112	0.003	0.109	0.103	0.003	0.100	0.110	0.003	0.107	1.071	1.224	1.067
R1_Ele1	0.000	0.000	0.000	0.000	0.000	0.000	0.000	0.000	0.000	0.000	0.000	0.000	0.000	0.000	0.000	0.000	0.000	0.000	1.104	1.112	1.096
RTE_Ele1-JAM1	0.094	0.006	0.089	0.072	0.007	0.065	0.091	0.004	0.087	0.061	0.004	0.058	0.083	0.006	0.077	0.076	0.004	0.072	0.918	0.666	0.938
Tc1_Ele7-MsqTc3	0.065	0.000	0.065	0.070	0.000	0.070	0.065	0.000	0.065	0.072	0.000	0.072	0.068	0.000	0.068	0.069	0.000	0.069	1.009	1.036	1.009
Ty1_copia_Ele56	0.292	0.155	0.137	0.179	0.079	0.100	0.394	0.174	0.220	0.263	0.114	0.149	0.236	0.117	0.119	0.329	0.144	0.184	1.394	1.232	1.555
Ty1_copia_Ele74	0.068	0.004	0.065	0.056	0.003	0.053	0.066	0.002	0.064	0.042	0.002	0.040	0.062	0.003	0.059	0.054	0.002	0.052	0.873	0.628	0.886
Ty1_copia_Ele122	0.044	0.002	0.042	0.056	0.003	0.053	0.039	0.001	0.038	0.039	0.001	0.038	0.050	0.002	0.048	0.039	0.001	0.038	0.781	0.550	0.791
Ty3_gypsy_Ele29	0.080	0.000	0.080	0.084	0.000	0.084	0.086	0.000	0.086	0.066	0.000	0.066	0.082	0.000	0.082	0.076	0.000	0.076	0.927	4.868	0.926
Ty3_gypsy_Ele49	0.019	0.004	0.016	0.024	0.004	0.020	0.019	0.004	0.015	0.020	0.003	0.017	0.022	0.004	0.018	0.019	0.003	0.016	0.892	0.877	0.895
Ty3_gypsy_Ele51	0.038	0.000	0.038	0.056	0.000	0.056	0.041	0.000	0.041	0.055	0.000	0.055	0.047	0.000	0.047	0.048	0.000	0.048	1.020	1.555	1.020
Ty3_gypsy_Ele52	0.040	0.000	0.040	0.065	0.000	0.064	0.051	0.000	0.051	0.060	0.000	0.060	0.053	0.000	0.052	0.056	0.000	0.055	1.058	0.877	1.059
Ty3_gypsy_Ele53	0.145	0.000	0.145	0.160	0.000	0.159	0.139	0.000	0.139	0.140	0.000	0.139	0.152	0.000	0.152	0.139	0.000	0.139	0.916	0.886	0.916
Ty3_gypsy_Ele54	0.240	0.001	0.239	0.170	0.000	0.170	0.239	0.000	0.239	0.150	0.000	0.150	0.205	0.000	0.204	0.195	0.000	0.194	0.950	0.574	0.951
Ty3_gypsy_Ele55	0.024	0.000	0.024	0.026	0.000	0.026	0.025	0.000	0.025	0.023	0.000	0.023	0.025	0.000	0.025	0.024	0.000	0.024	0.951	1.015	0.951
Ty3_gypsy_Ele57	0.039	0.000	0.039	0.041	0.000	0.041	0.039	0.000	0.039	0.033	0.000	0.033	0.040	0.000	0.040	0.036	0.000	0.036	0.905	1.027	0.905
Ty3_gypsy_Ele58	0.199	0.000	0.199	0.204	0.000	0.203	0.193	0.000	0.192	0.175	0.000	0.174	0.201	0.000	0.201	0.184	0.000	0.183	0.912	1.381	0.911
Ty3_gypsy_Ele59	0.348	0.000	0.347	0.354	0.000	0.353	0.328	0.000	0.328	0.300	0.000	0.300	0.351	0.000	0.350	0.314	0.000	0.314	0.896	0.982	0.896
Ty3_gypsy_Ele60	0.226	0.000	0.226	0.235	0.000	0.234	0.202	0.000	0.202	0.195	0.000	0.195	0.230	0.000	0.230	0.199	0.000	0.198	0.862	0.979	0.862
Ty3_gypsy_Ele61	0.049	0.000	0.049	0.067	0.000	0.067	0.050	0.000	0.050	0.051	0.000	0.051	0.058	0.000	0.058	0.051	0.000	0.051	0.871	1.307	0.870

Ty3_gypsy_Ele62	0.048	0.000	0.048	0.042	0.000	0.042	0.047	0.000	0.047	0.038	0.000	0.038	0.045	0.000	0.045	0.043	0.000	0.042	0.944	0.761	0.945
Ty3_gypsy_Ele73	0.273	0.056	0.217	0.409	0.040	0.369	0.304	0.047	0.257	0.483	0.026	0.457	0.341	0.048	0.293	0.394	0.037	0.357	1.155	0.758	1.220
Ty3_gypsy_Ele121	0.054	0.000	0.054	0.072	0.000	0.072	0.054	0.000	0.054	0.066	0.000	0.066	0.063	0.000	0.063	0.060	0.000	0.060	0.947	0.781	0.947
Ty3_gypsy_Ele122	0.238	0.001	0.237	0.291	0.001	0.289	0.247	0.001	0.246	0.264	0.001	0.263	0.264	0.001	0.263	0.255	0.001	0.254	0.966	0.944	0.966
Ty3_gypsy_Ele123	0.057	0.001	0.056	0.067	0.001	0.066	0.056	0.001	0.055	0.056	0.001	0.055	0.062	0.001	0.061	0.056	0.001	0.055	0.907	1.008	0.905
Ty3_gypsy_Ele124	0.012	0.000	0.012	0.012	0.000	0.012	0.013	0.000	0.013	0.012	0.000	0.012	0.012	0.000	0.012	0.012	0.000	0.012	1.034	1.663	1.034
Ty3_gypsy_Ele125	0.098	0.000	0.097	0.095	0.000	0.095	0.096	0.000	0.096	0.082	0.000	0.082	0.096	0.000	0.096	0.089	0.000	0.089	0.924	0.917	0.924
Ty3_gypsy_Ele126	0.198	0.002	0.197	0.216	0.001	0.215	0.187	0.001	0.185	0.174	0.001	0.173	0.207	0.001	0.206	0.180	0.001	0.179	0.870	0.955	0.870
Ty3_gypsy_Ele127	0.276	0.002	0.274	0.263	0.002	0.261	0.244	0.002	0.242	0.221	0.002	0.219	0.269	0.002	0.267	0.232	0.002	0.230	0.863	0.843	0.863
Ty3_gypsy_Ele128	0.232	0.000	0.232	0.254	0.000	0.254	0.217	0.000	0.216	0.238	0.000	0.238	0.243	0.000	0.243	0.228	0.000	0.227	0.935	0.972	0.935
Ty3_gypsy_Ele129	0.031	0.000	0.031	0.037	0.000	0.037	0.032	0.000	0.032	0.035	0.000	0.035	0.034	0.000	0.034	0.034	0.000	0.034	0.991	N/A	0.991
Ty3_gypsy_Ele132	0.041	0.000	0.041	0.048	0.000	0.048	0.048	0.000	0.048	0.054	0.000	0.054	0.044	0.000	0.044	0.051	0.000	0.051	1.146	0.000	1.146
Ty3_gypsy_Ele135	0.055	0.002	0.053	0.058	0.002	0.056	0.053	0.001	0.052	0.052	0.001	0.051	0.057	0.002	0.055	0.052	0.001	0.051	0.927	0.763	0.932
Ty3_gypsy_Ele137	0.055	0.000	0.055	0.051	0.000	0.051	0.053	0.000	0.053	0.043	0.000	0.043	0.053	0.000	0.053	0.048	0.000	0.048	0.906	1.044	0.906
Ty3_gypsy_Ele148	0.059	0.000	0.059	0.059	0.000	0.059	0.055	0.000	0.055	0.041	0.000	0.041	0.059	0.000	0.059	0.048	0.000	0.048	0.816	0.698	0.816
Ty3_gypsy_Ele152	0.197	0.000	0.197	0.212	0.000	0.212	0.195	0.000	0.194	0.196	0.000	0.196	0.205	0.000	0.205	0.195	0.000	0.195	0.953	0.882	0.953
Ty3_gypsy_Ele154	0.120	0.001	0.120	0.139	0.001	0.138	0.116	0.001	0.115	0.127	0.001	0.126	0.130	0.001	0.129	0.121	0.001	0.120	0.935	1.290	0.933
Ty3_gypsy_Ele155	0.636	0.001	0.635	0.614	0.002	0.612	0.541	0.001	0.540	0.503	0.001	0.502	0.625	0.001	0.623	0.522	0.001	0.521	0.836	0.918	0.836
Ty3_gypsy_Ele156	0.106	0.001	0.105	0.108	0.002	0.107	0.110	0.002	0.108	0.107	0.002	0.105	0.107	0.001	0.106	0.108	0.002	0.107	1.011	1.126	1.010
Ty3_gypsy_Ele158	0.094	0.000	0.093	0.149	0.000	0.149	0.093	0.000	0.092	0.130	0.000	0.130	0.121	0.000	0.121	0.111	0.000	0.111	0.916	0.720	0.916
Ty3_gypsy_Ele160	0.082	0.000	0.082	0.119	0.000	0.119	0.089	0.000	0.089	0.096	0.000	0.096	0.100	0.000	0.100	0.093	0.000	0.092	0.923	0.560	0.924
Ty3_gypsy_Ele163	0.064	0.000	0.063	0.082	0.000	0.082	0.082	0.000	0.081	0.085	0.000	0.085	0.073	0.000	0.072	0.083	0.000	0.083	1.147	1.168	1.147
Ty3_gypsy_Ele167	0.158	0.000	0.158	0.245	0.000	0.244	0.153	0.000	0.153	0.228	0.000	0.227	0.201	0.000	0.201	0.190	0.000	0.190	0.946	1.399	0.946
Ty3_gypsy_Ele171	0.048	0.000	0.048	0.051	0.000	0.051	0.041	0.000	0.041	0.039	0.000	0.039	0.049	0.000	0.049	0.040	0.000	0.040	0.810	0.835	0.810
